# Dynamic early biomarkers predict outcomes in pediatric Epstein–Barr virus‐associated hemophagocytic lymphohistiocytosis

**DOI:** 10.1002/ped4.70071

**Published:** 2026-08-18

**Authors:** Feifei Liu, Qing Zhang, Sitong Chen, Yunze Zhao, Dong Wang, Hongyun Lian, Honghao Ma, Tianyou Wang, Rui Zhang, Zhigang Li

**Affiliations:** ^1^ Laboratory of Hematologic Diseases Beijing Pediatric Research Institute Beijing Children's Hospital Capital Medical University National Center for Children's Health Beijing China; ^2^ National Key Discipline of Pediatrics Capital Medical University Beijing China; ^3^ Key Laboratory of Major Diseases in Children Ministry of Education Beijing China; ^4^ Department of Hematology Beijing Children's Hospital Capital Medical University National Center for Children's Health Beijing China

**Keywords:** Children, Cytokine, Early biomarkers, Epstein–Barr virus‐associated hemophagocytic lymphohistiocytosis, Ferritin, Interferon‐γ, Outcomes

## Abstract

**Importance:**

Pediatric Epstein–Barr virus (EBV)‐associated hemophagocytic lymphohistiocytosis (HLH) is a life‐threatening disorder, and early identification of poor responders is critical to improving survival. However, no convenient clinical tools exist to predict individual prognosis using dynamic biomarkers during early treatment.

**Objective:**

To investigate the predictive value of early dynamic changes in plasma biomarkers and to develop a prognostic model for children with EBV‐HLH.

**Methods:**

This retrospective study enrolled 60 newly diagnosed pediatric EBV‐HLH patients. We analyzed the longitudinal changes in plasma EBV‐DNA (pEBV‐DNA), ferritin, and cytokine levels during the etoposide‐based induction first‐line therapy. Independent prognostic factors were identified using Cox multivariate regression and LASSO, followed by the construction of a prognostic nomogram. Model performance was evaluated through area under the curve (AUC), decision curve analysis, and internal cross‐validation.

**Results:**

Multivariate analysis identified positive pEBV‐DNA at week 2 (w2) and low decreases in ferritin (ΔFerritin.w2) and interferon (IFN)‐γ (ΔIFN‐γ.w2) as independent predictors of adverse outcomes. A nomogram integrating these three variables demonstrated a superior AUC of 0.834 (vs. 0.677 for pEBV‐DNA.w2 alone, *P* = 0.003). The model successfully stratified patients into low‐ and high‐risk groups with significantly different 3‐year event‐free survival (84.8% vs. 33.3%, *P* < 0.001).

**Interpretation:**

The proposed model, based on dynamic plasma biomarkers, provides a promising tool for the early risk stratification of pediatric EBV‐HLH. Composed of pEBV‐DNA, ΔFerritin, and ΔIFN‐γ at w2, this nomogram can effectively identify patients at high risk of first‐line treatment failure. Given the exploratory nature of this study, these findings require further validation in larger, independent cohorts.

## Graphical abstract

This study elucidates the critical pathogenic mechanisms of pediatric Epstein–Barr virus‐associated hemophagocytic lymphohistiocytosis (EBV‐HLH), where EBV infection triggers a cytokine storm leading to immune cell hyperactivation and tissue damage. Dynamic plasma biomarkers (EBV‐DNA, ferritin, and interferon‐γ) effectively predict treatment response and prognosis.

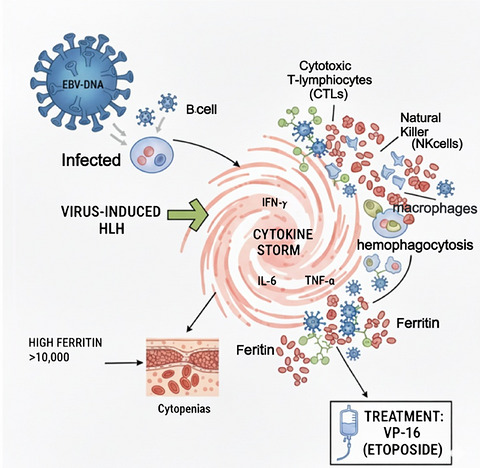

## INTRODUCTION

Hemophagocytic lymphohistiocytosis (HLH) is a life‐threatening hyperinflammatory disorder characterized by dysregulated immune activation leading to the uncontrolled proliferation of lymphocytes and macrophages, culminating in a cytokine storm.^1^, ^2^ HLH predominantly affects children and adolescents and is classified into primary HLH (pHLH) and secondary HLH (sHLH).^3^ Infection‐related HLH is the main type of sHLH, particularly Epstein–Barr virus–associated HLH (EBV‐HLH), which accounts for 74.4% of pediatric HLH cases in China.^4^

EBV infection can present as infectious mononucleosis (IM), chronic active EBV disease (CAEBVD), EBV‐HLH, or EBV‐related autoimmune diseases.^5^ Notably, the incidence of EBV‐HLH is disproportionately high in Asian populations and represents the most severe clinical manifestation of EBV‐associated diseases, with the mortality rate approaching 40%.^6^

The HLH‐94 regimen, developed by the Histiocyte Society in 1994, along with its refined version HLH‐04, is based on etoposide and dexamethasone and is the most commonly used therapeutic approach for EBV‐HLH.^7^ Approximately 30%–40% of patients with EBV‐HLH still fail to respond to etoposide‐based therapy.^8^ Additionally, a heightened frequency of disease recurrence is observed at weeks 5–8 of induction therapy consisting of weekly etoposide administration alongside tapered dexamethasone dosing.^6^, ^9^, ^10^ Inadequate responses prompt consideration of either reintensifying etoposide/dexamethasone or switching salvage therapy. Early and precise assessment of the response to induction therapy enables the identification of poor responders with unfavorable prognoses.^11^ While studies have identified various pretreatment markers at diagnosis that correlate with mortality, their usefulness is limited by an inability to account for the variable and dynamic responses to initial therapy.^12^

In this study, we conducted an analysis of clinical and laboratory data from children with EBV‐HLH treated with etoposide‐based first‐line regimens. This study was designed to develop a predictive model for therapeutic response and prognosis, allowing the early identification of children at risk for treatment failure to guide clinically adaptive therapeutic interventions in the effort to improve survival rates.

## METHODS

### Ethical approval

This study was approved by the Ethics Committee of Beijing Children's Hospital (No.2025‐E‐186‐R) and complied with the Declaration of Helsinki.

### Data collection

A retrospective analysis was conducted on 60 children with newly diagnosed EBV‐HLH who were admitted to Beijing Children's Hospital, Capital Medical University, between June 2017 and September 2020. The inclusion criteria were as follows: (1) age < 18 years at diagnosis, (2) met the HLH‐2004 diagnostic criteria and the diagnostic criteria for EBV‐HLH, and (3) received etoposide‐based first‐line chemotherapy as the initial treatment. The exclusion criteria included primary HLH, malignancy‐associated HLH, EBV reactivation‐associated HLH (such as CAEBVD‐HLH), or prior immunotherapy or chemotherapy.

### Diagnostic criteria

The diagnosis of HLH was performed in accordance with the HLH‐2004 regimen, and the criteria^13^ are listed in Table S1. Central nervous system (CNS) involvement was defined according to HLH‐2004 criteria as the presence of at least one of the following: 1) clinical neurological symptoms (e.g., seizures and altered consciousness); 2) abnormal cranial magnetic resonance imaging (MRI) findings; 3) cerebrospinal fluid (CSF) abnormalities (white blood cell count > 5 × 10^6^/L or protein > 0.5 g/L).

The diagnosis of EBV‐HLH must be accompanied by evidence of EBV infection: 1) serologic antibody assays indicative of primary acute or active EBV infection (Primary EBV infection was defined as positive viral capsid antigen‐IgM and negative Epstein‐Barr nuclear antigen‐immunoglobulin G (EBNA‐IgG) results. Patients with positive EBNA‐IgG were excluded to rule out past or chronic infection; 2) molecular methods, including polymerase chain reaction (PCR) or in situ Southern hybridization, demonstrating EBV positivity in serum, bone marrow, lymph nodes, or other affected tissues; 3) exclusion of CAEBVD, malignancy, and autoinflammatory disorders.

### Treatment and evaluation

All 60 children received first‐line chemotherapy based on etoposide after diagnosis, which was administered in two distinct phases:

1) Induction therapy (8 weeks): Dexamethasone [10 mg/(m^2^·d), tapered to discontinuation] and etoposide (VP‐16: 100 mg/m^2^ twice weekly during the initial 2 weeks and then weekly thereafter). 2) Maintenance therapy (9–40 weeks): Dexamethasone [10 mg/(m^2^·d) × 3 d biweekly], etoposide (VP‐16; 100 mg/m^2^ biweekly), and cyclosporin A [CSA: 6 mg/(kg·d) until treatment completion].

Patients who did not respond or who experienced relapse during treatment were treated with salvage regimens. Salvage regimens included the DEP regimen (liposomal doxorubicin + etoposide + methylprednisolone), L‐DEP regimen (polyethylene glycol‐conjugated asparaginase + DEP), antithymocyte globulin, and anti‐CD52 monoclonal antibody.^14^, ^15^

Treatment response was evaluated at the following time points: Day 3, the end of week (w)1, w2, w4, and w8 of induction therapy; and additional time points if necessary. Responses were defined as complete response (CR), no response (NR), or relapse (Table S2).^16^, ^17^ The patients were divided into two groups according to the first‐line treatment outcome: favorable group (sustained CR following the first‐line treatment) and unfavorable group (NR, relapse, or death from any cause).

For patients with EBV‐HLH, the decision to discontinue treatment should be individualized based on the patient's clinical course and the achievement of a complete clinical response.

### Clinical and laboratory data

Clinical and laboratory data were collected at diagnosis. In addition, the plasma levels of EBV‐DNA, ferritin, and disease‐related cytokines [interferon (IFN)‐γ, interleukin (IL)‐10, and IL‐6] ^18^ were measured on day 3 and at the end of w1, w2, and w4 of induction therapy.

Soluble CD25 (sCD25) was quantified by ELISA (Human sCD25 ELISA Kit; Multi Sciences, China). Serum ferritin levels were measured using a chemiluminescence kit (Access Ferritin, 33020; Beckman, USA). The plasma EBV‐DNA (pEBV‐DNA) load was detected by a PCR‐fluorescent probe kit (DaAn Gene Co., Guangzhou, China).^17^ Cytokine levels were determined using flow cytometry (Human Th1/Th2 Cytokine Kit II, 551809; BD Biosciences, San Jose, California).^18^

### Statistical analyses

The follow‐up period ended on December 31, 2024, and the median follow‐up duration was 68.0 months (interquartile range, 32.1–83.9 months). Event‐free survival (EFS) was defined as the time from diagnosis to NR, relapse, or death from any cause, whichever came first, or the last contact with the patient. We analyzed the data using R (v4.2.1) for Generalized Estimating Equations (GEE) of biomarkers and IBM SPSS 27.0 for all other statistics. Univariate and multivariate Cox proportional hazards regression models were used to assess the associations between the clinical/laboratory indices and prognosis. The proportional hazards assumption was verified using Schoenfeld residual tests (Table S3), and model stability was assessed via multicollinearity and influential observation diagnostics. LASSO Cox regression was applied to identify the optimal predictors. A multivariate Cox proportional hazards model was then constructed using these optimal predictors to predict EFS. A nomogram was developed on the basis of the regression coefficients of this Cox model. Missing data rates were below 5% for all main predictors (Table S4), so we used complete‐case analysis for the Cox regression and nomogram. The model's predictive performance was evaluated using time‐dependent calibration curves (e.g., for 1‐, 2‐, and 3‐year EFS) to assess the agreement between predicted and observed outcomes. Decision curve analysis (DCA) was used to determine the model's clinical net benefit. Internal validation of the model was performed through cross‐validation on the training set. The Kaplan‐Meier method was used to estimate EFS, and the log‐rank test was used to compare outcomes between different patient groups. A receiver operating characteristic (ROC) curve was used to evaluate the ability of biomarker changes to predict the treatment outcomes of first‐line therapy, and the prognostic value of the nomogram was assessed. The threshold for statistical significance was set at *P* < 0.05.

## RESULTS

### Patient characteristics

This study included 60 children with EBV‐HLH. The cohort comprised 37 males and 23 females, with a male‐to‐female ratio of 1.61:1. The median patient age was 3.1 years (range, 1.7–6.4 years). Eighteen patients (30.0%) were under two years old. A total of 47 patients (78.3%) had fever with a duration of more than 7 days. Other common findings included hepatomegaly (85.0%), splenomegaly (93.3%), lymphadenopathy (95.3%), hemophagocytosis in the bone marrow (91.7%), and CNS involvement (26.7%). Among the 16 patients with CNS involvement, 10 (62.5%) had abnormal MRI findings. Of these, 5 had isolated MRI abnormalities, 2 had additional clinical symptoms, 2 had additional CSF abnormalities, and 1 had all three. Of the remaining 6 patients without MRI abnormalities, 1 (6.3%) presented with clinical symptoms alone, and 5 (31.3%) were diagnosed based on CSF abnormalities alone. Notably, all patients who died during the follow‐up period were classified into the unfavorable treatment outcome group, as their death occurred following non‐response or disease relapse (Table 1). The 1‐year and 3‐year EFS rates for the entire cohort were 80.0% ± 5.2% and 73.3% ± 5.7%, respectively. The 1‐year OS rate was 94.7% ± 3.0%.

**TABLE 1:** Characteristics of the patients included in this study

		First‐line treatment outcome		
Variables	Total *n* = 60	Favorable *n* = 44	Unfavorable *n* = 16	*P*
Gender				0.936
Male	37 (61.7)	27 (61.4)	10 (62.5)	
Female	23 (38.3)	17 (38.6)	6 (37.5)	
Age (at diagnosis, years)	3.1 (1.7, 6.4)	3.3 (2.0, 6.5)	2.5 (1.3, 4.2)	0.324
Survival				**0.013**
Live	57 (95.0)	44 (100.0)	13 (81.2)	
Death	3 (5.0)	0 (0.0)	3 (18.8)	
Fever (day)				0.481
< 7	13 (21.7)	11 (25.0)	2 (12.5)	
≥ 7	47 (78.3)	33 (75.0)	14 (87.5)	
Hepatomegaly				1.000
No	9 (15.0)	7 (15.9)	2 (12.5)	
Yes	51 (85.0)	37 (84.1)	14 (87.5)	
Splenomegaly				0.565
No	4 (6.7)	4 (9.1)	0 (0.0)	
Yes	56 (93.3)	40 (90.9)	16 (100.0)	
Lymphadenopathy				1.000
No	3 (5.0)	2 (4.5)	1 (6.2)	
Yes	57 (95.0)	42 (95.5)	15 (93.8)	
Hemophagocytosis				1.000
No	5 (8.3)	4 (9.1)	1 (6.2)	
Yes	55 (91.7)	40 (90.9)	15 (93.8)	
CNS involvement				1.000
No	44 (73.3)	32 (72.7)	12 (75.0)	
Yes	16 (26.7)	12 (27.3)	4 (25.0)	
WBC (×10^9^/L)	3.5 (2.1, 6.7)	3.8 (2.2, 7.0)	3.0 (1.6, 6.2)	0.467
Hb (g/L)	93.3 ± 17.0	95.8 ± 17.9	86.5 ± 12.5	0.059
Plt (×10^9^/L)	64.0 (42.0, 104.5)	71.0 (43.0, 113.0)	53.0 (33.2, 81.0)	0.235
ALT (U/L)	127.1 (48.5, 237.0)	152.6 (84.1, 241.3)	54.0 (23.3, 145.4)	**0.034**
AST (U/L)	211.4 (108.5, 410.4)	237.6 (146.0, 414.7)	136.2 (83.1, 397.9)	0.137
TG (mmol/L)	3.3 ± 2.0	3.3 ± 2.0	3.2 ± 2.1	0.864
LDH (U/L)	1099.0 (679.8, 2363.2)	1170.5 (679.8, 2379.8)	1074.5 (672.8, 2315.2)	0.867
Fib (g/L)	1.5 ± 0.6	1.5 ± 0.6	1.7 ± 0.7	0.258
D‐Dimer (mg/L)	3.2 (1.3, 5.7)	3.2 (1.7, 6.4)	3.0 (0.9, 4.2)	0.483
sCD25 (pg/mL)	31607.0 (22411.8, 41742.2)	31857.5 (19675.5, 42159.0)	30510.0 (26058.5, 35317.5)	0.993
Ferritin (ng/mL)	3679.0 (1282.9, 11023.2)	6392.5 (1616.5, 11797.2)	2001.0 (663.6, 5138.8)	0.124
pEBV‐DNA status				0.311
Negative	29 (48.3)	23 (52.3)	6 (37.5)	
Positive	31 (51.7)	21 (47.7)	10 (62.5)	
IFN‐γ (pg/mL)	41.0 (6.2, 175.8)	36.9 (3.0, 175.8)	56.0 (20.0, 174.7)	0.389
IL‐6 (pg/mL)	39.4 (9.1, 93.6)	34.5 (9.4, 93.6)	49.6 (8.9, 89.3)	0.701
IL‐10 (pg/mL)	78.3 (16.0, 403.1)	78.3 (16.0, 366.1)	79.0 (17.5, 436.4)	0.973

Continuous data are presented as mean ± standard deviation for normally distributed variables and median (interquartile range) for non‐normally distributed variables. Categorical variables are expressed as *n* (%).

Abbreviations: ALT, alanine aminotransferase; AST, aspartate aminotransferase; CNS, central nervous system; EFS, event‐free survival; Hb, hemoglobin; OS, overall survival; Plt, platelet count; TG, triglyceride; LDH, lactate dehydrogenase; Fib, fibrinogen; sCD25, soluble cluster of differentiation 25; pEBV‐DNA, plasma Epstein–Barr virus DNA; IFN‐γ, interferon‐gamma; IL, interleukin; WBC, white blood cell count.

### Relationships between early dynamic plasma biomarkers and the outcome of first‐line therapy

**FIGURE 1:**
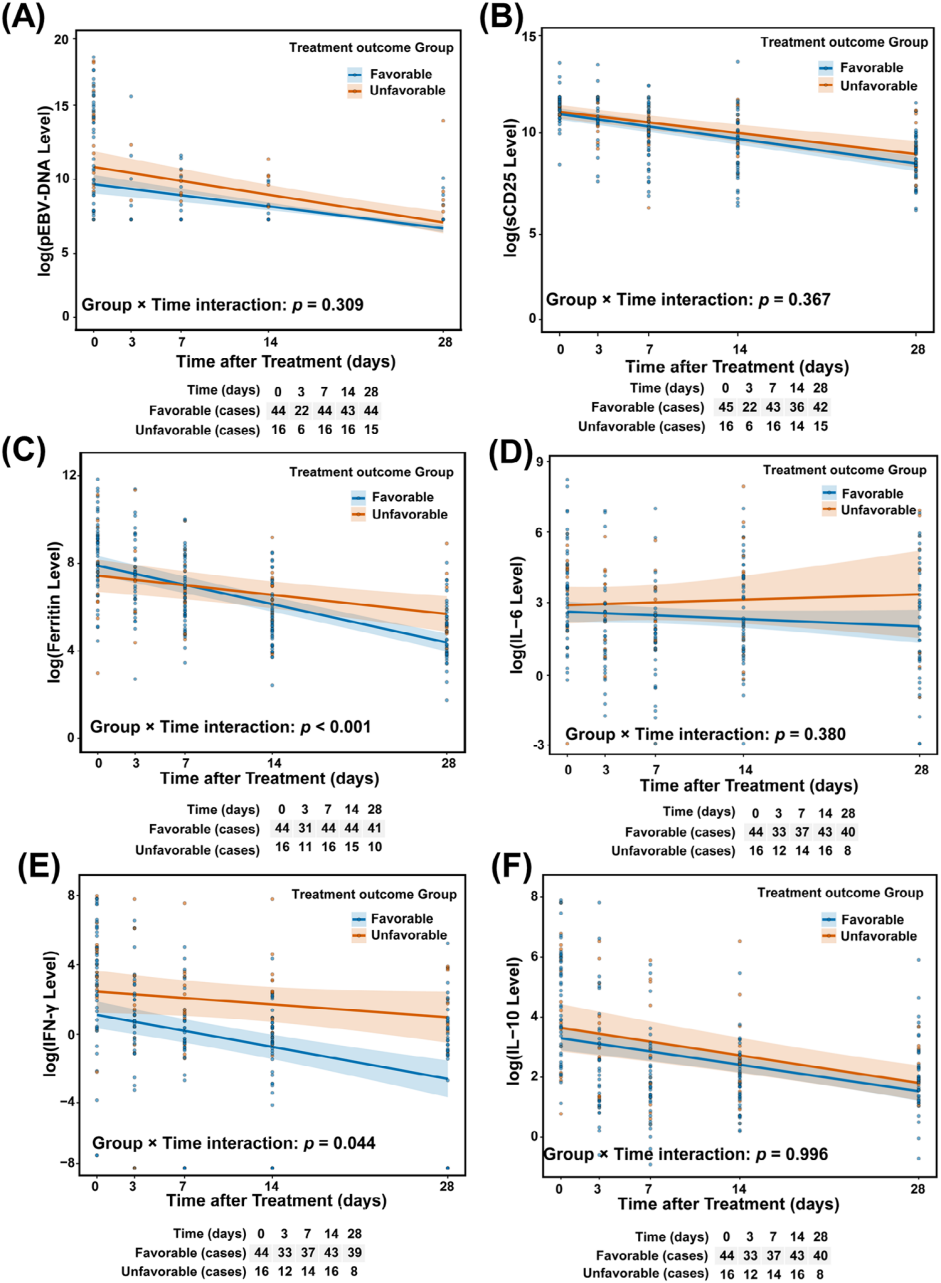
Longitudinal changes in plasma biomarker levels (log‐transformed) assessed by Generalized Estimating Equations (GEE) for the favorable versus unfavorable treatment outcome groups. Statistically significant differences in the Group × Time interaction effect were observed for ferritin (C) and IFN‐γ (E) (*P* < 0.05). In contrast, no significant differences were found for pEBV‐DNA (A), sCD25 (B), IL‐6 (D), or IL‐10 (F) (*P* > 0.05). Data are shown as log10 (level+1) to accommodate zero values. *P* for group×time interaction from GEE.

We first analyzed the levels of sCD25, ferritin, pEBV‐DNA, IL‐10, IL‐6, and IFN‐γ in the favorable treatment outcome and unfavorable treatment outcome groups at multiple time points (before treatment, day 3, and at the end of w1, w2, and w4). The trends were visualized using GEE curves (Figure 1).

While the biomarker levels were generally higher in the favorable treatment outcome group, significant group‐by‐time interactions were found only for ferritin and IFN‐γ (group × time *P* < 0.001, and *P* = 0.044, respectively). These findings indicate that the dynamic changes in these two markers over time differed significantly between the two groups. In contrast, no such differences were observed for pEBV‐DNA, sCD25, IL‐6, or IL‐10 (*P* > 0.05).

### Association between changes in pEBV‐DNA, ferritin, and IFN‐γ during initial chemotherapy and treatment outcomes

To assess their prognostic value, we analyzed early changes in pEBV‐DNA, ferritin, IFN‐γ, and other clinical indicators. The relative changes (Δ) in ferritin and IFN‐γ levels were calculated as [(level at the end of w1 or w2‐diagnostic level)/diagnostic level]. As shown in Table S5, patients in the favorable group exhibited significantly greater relative decreases in both biomarkers compared to those in the unfavorable group. Subsequently, ROC analysis revealed significant associations between treatment response and ΔFerritin [w1 & w2] and ΔIFN‐γ (w1 & w2) (Figure S1). Patients were grouped by cutoff (−0.651 and −0.935 for ΔFerritin and −0.711 and −0.981 for ΔIFN‐γ at the end of w1 and w2, respectively) into “low‐decrease” (Δ > cutoff) and “high‐decrease” (Δ < cutoff) groups using Youden's index. Patients were also grouped by the pEBV‐DNA load at the end of w1 and w2 into negative (< 500 copies/mL) and positive (≥ 500 copies/mL) groups.

Cox univariate analysis (Table S6) revealed that positive pEBV‐DNA, a low decrease in ∆Ferritin at the end of w1 and w2, a low decrease in ∆IFN‐γ at the end of w2, and positive pEBV‐DNA at the end of w2 were significantly associated with poor prognosis (all *P* < 0.05). No correlation was observed between other clinical features and patient prognosis (*P* > 0.05).

**FIGURE 2:**
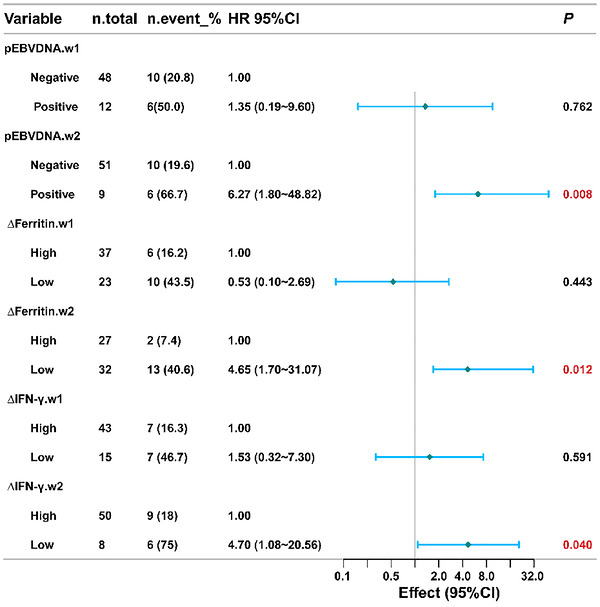
Forest plot of the multivariate Cox regression analysis. Low: low‐decrease; High: high‐decrease; HR, hazard ratio. Positive plasma Epstein–Barr virus‐DNA (pEBV‐DNA) and low decreases of ∆Ferritin and ∆IFN‐γ at w2 were statistically significant (*P* < 0.05).

In the Cox multivariate analysis (Figure 2), three factors emerged as independent predictors of poor outcomes: positive pEBV‐DNA.w2 (HR: 6.27[1.80–48.82]; *P* = 0.008), a low decrease in ∆Ferritin.w2 (HR: 4.65[1.70–31.07]; *P* = 0.012), and a low decrease in ∆IFN‐γ.w2 (HR: 4.70[1.08–20.56]; *P* = 0.040).

### Risk scoring model

**FIGURE 3:**
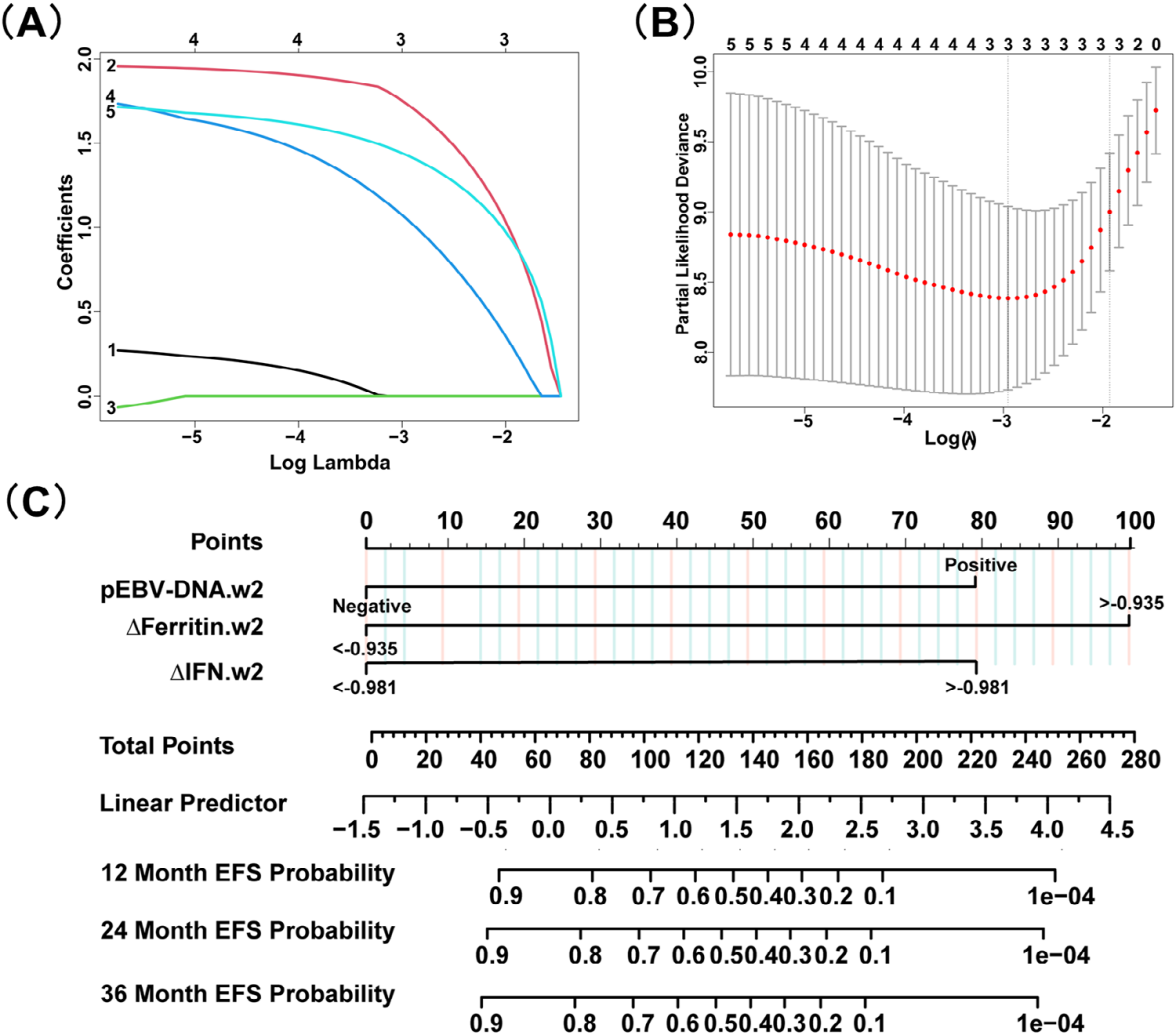
Variable selection and the prognostic nomogram. (A) LASSO coefficient paths. This plot shows how the variables were selected by shrinking coefficients. (B) Selection of the best lambda (λ). 10‐fold cross‐validation was used to find the best model. The dotted line marks the chosen value. (C) Nomogram to predict EFS. To estimate survival probability, add the points for the three variables [plasma Epstein–Barr virus‐DNA (pEBV‐DNA), ΔFerritin, and ΔIFN‐γ at w2] to get the “Total Points”. Cutoffs derived from Youden's index of receiver operating characteristic curves (see Figure S1). EFS, event‐free survival.

We used LASSO Cox regression to select the optimal set of predictors from multiple variables. The model with three variables, pEBV‐DNA.w2, ∆Ferritin.w2, and ∆IFN‐γ.w2, demonstrated the best predictive performance and stability (Figure 3A,B). These variables matched those identified by Cox regression analysis, which reinforces the reliability of our findings.

The prognostic nomogram (Figure 3C) was constructed based on 58 patients with complete data (Table S4) using these three selected variables: pEBV‐DNA.w2, ∆Ferritin.w2, and ∆IFN‐γ.w2. The model assigns points to each variable on the basis of its regression coefficient; summing these points gives a total score, which is then converted into the probability of an adverse outcome.

**FIGURE 4:**
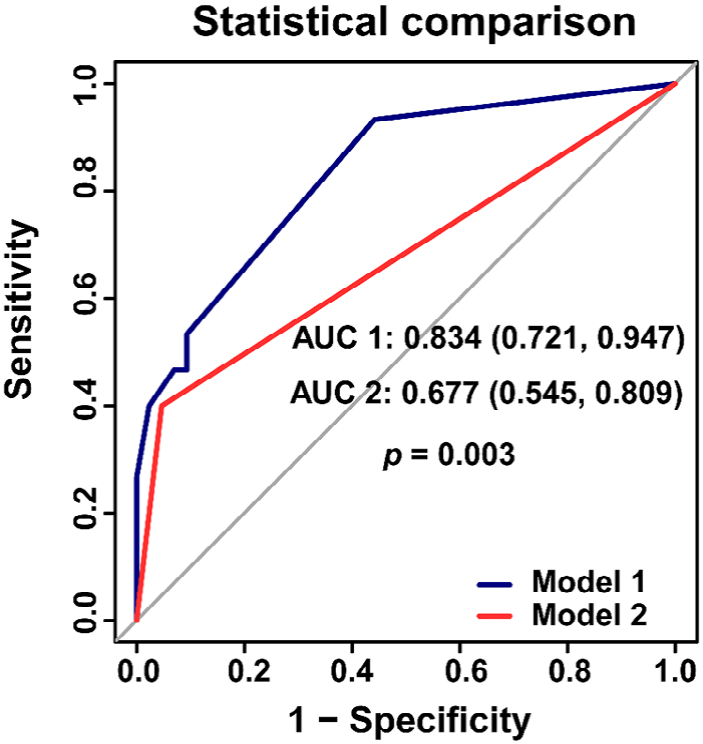
Statistical comparison of the predictive performance between the two models. The receiver operating characteristic (ROC) curves evaluate the discriminatory ability of two models for predicting treatment outcomes in pediatric EBV‐HLH. Model 1: pEBV‐DNA.w2 + ΔFerritin.w2 + ΔIFN‐γ.w2; Model 2: pEBV‐DNA.w2*. P*‐value from DeLong's test for two correlated ROC curves. w2, at the end of week 2; pEBV‐DNA, plasma Epstein–Barr virus‐DNA.

We compared our model's performance to that of the pEBV‐DNA load at the end of w2, which is a commonly used clinical index. According to the results of the ROC curve analysis, the area under the curve (AUC) of the nomogram was 0.834, which was significantly greater than that of 0.677 for the pEBV‐DNA load (Figure 4; *P* = 0.003).

**FIGURE 5:**
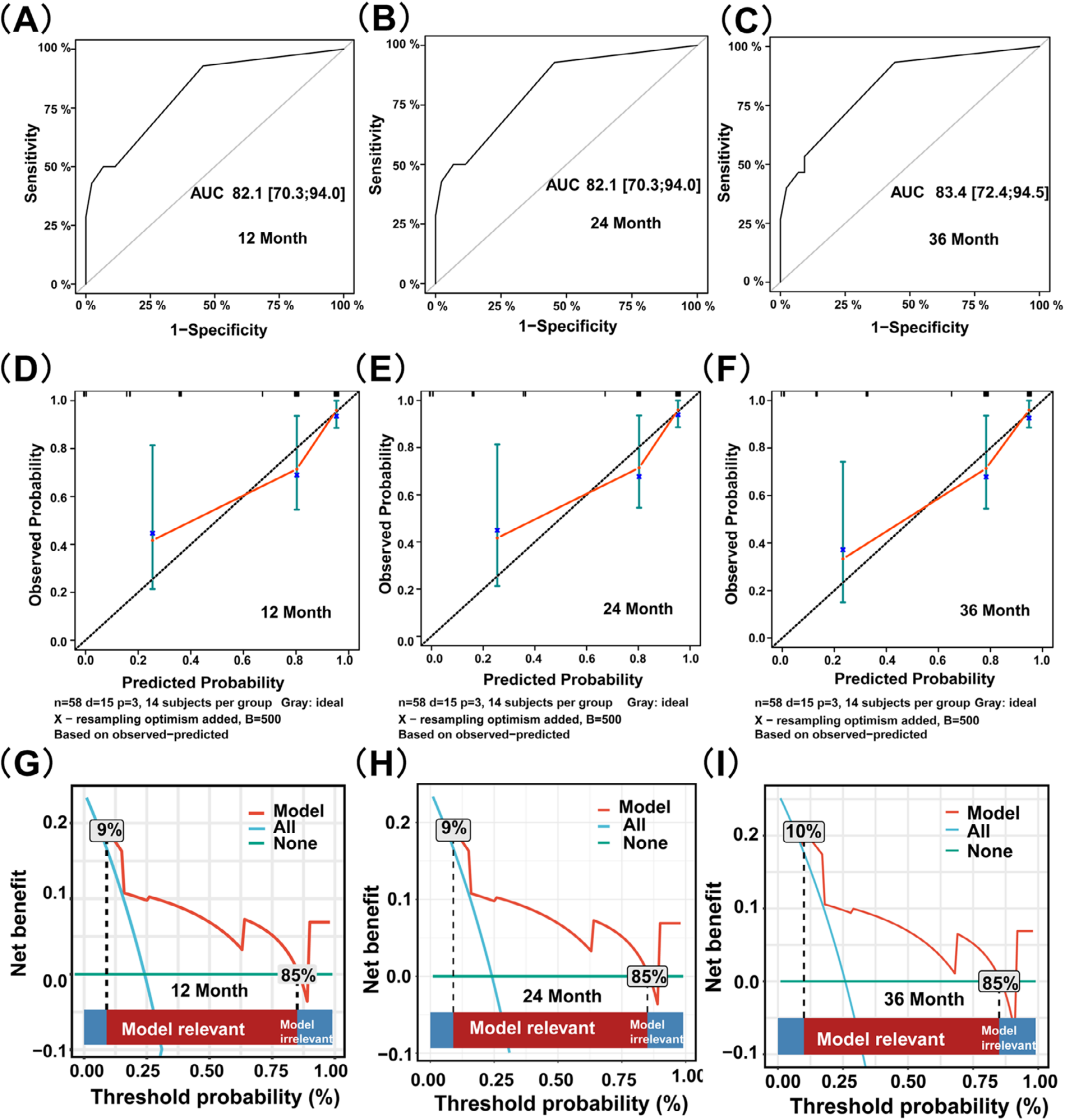
Performance assessment of the prognostic model for pediatric Epstein–Barr virus‐associated hemophagocytic lymphohistiocytosis. (A–C) Time‐dependent receiver operating characteristic curves evaluating the model's discriminatory ability at 12, 24, and 36 months, respectively. (D–F) Calibration curves demonstrating the agreement between predicted and observed survival probabilities at 12, 24, and 36 months, with bootstrap resampling (*n* = 500). The gray dashed line represents the ideal model, and the orange solid line represents the performance of the model. (G–I) Decision curve analysis (DCA) evaluating the clinical net benefit of the model at 12, 24, and 36 months. The red line represents the model, the blue line represents the strategy of treating all patients, and the green line represents the strategy of treating no patients.

We assessed the model's performance with internal cross‐validation. The predictive accuracy of our model was validated over time. The time‐dependent AUC values were 0.821, 0.821, and 0.834 at 12, 24, and 36 months, respectively (Figure 5A–C), with a bias‐corrected Dxy statistic of 0.625 and a mean AUC of 0.824. The calibration was also excellent (Figure 5D–F), with a slope of 0.811 and a mean Brier score of 0.145, indicating strong agreement between the predicted and observed survival outcomes.

DCA (Figure 5G–I) confirmed the clinical utility of the model by demonstrating a greater net benefit than alternative strategies did. Using the model's risk score, we stratified patients into low‐ and high‐risk groups. Compared with the high‐risk group, the low‐risk group had a significantly better 3‐year EFS (84.8% ± 5.3% vs. 33.3% ± 15.1%, *P* < 0.001; Figure S2).

## DISCUSSION

EBV‐HLH is the most common form of secondary HLH; however, its pathogenesis remains unclear.^19^ Numerous studies have shown that EBV infects CD8^+^ T lymphocytes and natural killer cells. This leads to overactivation of the lymphoid tissue and consequently to a cytokine storm. Therefore, the clinical symptoms vary among patients. In this study, we observed that the main clinical features were prolonged fever and hepatomegaly, splenomegaly, and lymphadenopathy. Notably, more than 25% of the children had central nervous system involvement, which needs close attention.

Currently, the standard therapy for EBV‐HLH is the HLH‐94 or HLH‐04 chemotherapy regimen, which is based on etoposide and dexamethasone.^20^ According to the results of the HLH‐2004 study, this combination therapy can save most patients but does not significantly improve their long‐term outcomes.^6^ Studies report that the overall mortality rate for children with EBV‐HLH can be as high as 61.4% within three years.^21^ Identifying patients who respond poorly to treatment during initial therapy could enable timely escalation to salvage regimens. While the HScore^22^, ^23^, ^24^ provides risk stratification in adults with HLH, no validated tool exists for prognosticating pediatric EBV‐HLH either at diagnosis or during early treatment.

Studies on EBV‐related diseases have shown that the pEBV‐DNA load before and after treatment can help predict patient survival.^25^ This load is often used as a minimally invasive biomarker for screening or evaluating treatment response in a range of EBV‐related malignancies.^26^ Some studies have suggested that a high pEBV‐DNA load in T cells may serve as a potential predictor of the progression of EBV‐HLH.^27^ Consistent with these observations, this study revealed that pEBV‐DNA loads, especially at the end of w1 and w2, were strongly linked to the response to 1st‐line therapy and to patient outcomes. Nevertheless, despite its clinical utility, pEBV‐DNA alone is insufficient to reflect a patient's immune status or therapeutic efficacy, as EBV acts as a trigger of disease, and individual immune responses vary widely. Other clinical indicators are needed to comprehensively assess pEBV‐DNA levels.

Many previous studies have indicated that some specific cytokines (IFN‐γ, IL‐6, and IL‐10) could be useful biomarkers for the diagnosis, differential diagnosis, and surveillance of EBV‐HLH.^28^, ^29^, ^30^ Jordan's team reported the serial monitoring of sCD25 during etoposide‐based therapy for HLH to be the most potent single predictor of mortality prior to bone marrow transplantation.^30^ Similarly, a high serum ferritin level is helpful for diagnosing HLH, and a decrease in the serum ferritin level often indicates effective treatment.^31^, ^32^, ^33^ Owing to the use of various detection methods and the absence of a unified international standard,^34^ utilizing the relative change from baseline may serve as an effective alternative. In this study, we found that positive pEBV‐DNA.w2 and low decreases in both ∆ferritin.w2 and ∆IFN‐γ.w2 were independently related to poor prognosis. We used these three factors to construct a nomogram to facilitate the prediction of patient outcome and the identification of patients at high risk of treatment failure. This identification could then support the decision to pursue alternative or salvage treatment.

Previous studies have demonstrated that sCD25 plays an important role in the prognosis evaluation of HLH.^35^, ^36^ However, we observed only a weak link between the sCD25 level or change and patient outcome in our patients. Differences in disease subtypes, patient ages, and ethnic backgrounds could contribute to these results. Moreover, the detection methods and thresholds of sCD25 vary among different institutions. Future studies with large sample sizes and standardized detection methods are needed to validate our results.

This study included only newly diagnosed patients who received standardized etoposide‐based therapy. HLH is a rare disease, and children with EBV‐HLH often receive various treatments at other hospitals; newly diagnosed patients are uncommon in our center. Therefore, the small sample size in this study led to the risk of overfitting. In addition, continuous biomarker changes were dichotomized using Youden's index derived from ROC analyses, which facilitates clinical application but discards information, reduces power, and can lead to optimistic estimates. The events‐per‐variable ratio in this study was below recommended thresholds. Thus, the exploratory model developed in this study could be improved using continuous biomarker changes and requires further validation through prospective, large‐sample studies.

The present results demonstrated that dynamic plasma markers had predictive value for outcomes of EBV‐HLH patients following treatment with etoposide‐based therapy. The model comprising early (w2) response pEBV‐DNA, ∆Ferritin, and ∆IFN‐γ could predict therapeutic response and prognosis in pediatric EBV‐HLH patients.

## CONFLICT OF INTEREST

The authors declare no conflict of interest.

## Supporting information



Supporting Information
