# Supplementary material for: Dynamic early biomarkers predict outcomes in pediatric Epstein–Barr virus‐associated hemophagocytic lymphohistiocytosis
Source: Pediatr Investig. 2026 Aug 18:10.1002/ped4.70071. Online ahead of print. doi: 10.1002/ped4.70071 (PMC13483129; doi:10.1002/ped4.70071)
Supplement: Supplementary file 1 [file PED4-9999-0-s001.pdf]

Supplementary Material for

Dynamic early biomarkers predict outcomes in pediatric Epstein–Barr virus-associated hemophagocytic lymphohistiocytosis

Feifei Liu, Qing Zhang, PhD, Sitong Chen, Yunze Zhao, Dong Wang, Hongyun Lian, Honghao Ma, Tianyou Wang, Rui Zhang, Zhigang Li

TABLE S1 Diagnostic criteria for HLH used in the HLH-2004 trial*

**The diagnosis of HLH may be established:**
**A.Molecular diagnosis consistent with HLH: pathologic mutations of *PRF1, UNC13D, Munc18-2, Rab27a, STX11, SH2D1A, or BIRC4***
or
**B. Five of the 8 criteria listed below are fulfilled:**
1.Fever ≥ 38.5 ℃
2.Splenomegaly
3.Cytopenias (affecting at least 2 of 3 lineages in the peripheral blood)
Hemoglobin < 9 g/dL(in infants < 4weeks: hemoglobin < 10 g/dL)
Platelets <100 × 10^9/L
Neutrophils < 1 × 10^9/L
4.Hypertriglyceridemia (fasting, > 3.0 mmol/L) and/or hypofibrinogenemia(< 1.5 g/L)
5.Hemophagocytosis in bone marrow, spleen, lymph nodes, or liver
6.Low or absent NK-cell activity
7.Ferritin > 500 ng/mL
8.Elevated sCD25 (α-chain of sIL-2 receptor)**

* Cited from Michael B. Jordan et al.

**Elevations above age-adjusted, laboratory-specific normal levels (defined as 2 SD from the mean) appear more meaningful than the original designation of 2400 U/mL because of variations between laboratories.

HLH, hemophagocytic lymphohistiocytosis.

TABLE S2 EBV-HLH therapeutic response assessment criteria

Category	Application of criteria
Complete Response	Resolution of all quantifiable clinical symptoms, signs, and laboratory abnormalities attributable to HLH, meeting all of the following criteria:<br>1) No fever: body temperature < 37.5℃.<br>2) Normal spleen size on abdominal ultrasound.<br>3) No cytopenias without blood product transfusion: absolute neutrophil count ≥ 1.0×10^9/L, platelet count ≥ 100×10^9/L, hemoglobin ≥ 110 g/L.<br>4) Fibrinogen > 1.50 g/L.<br>5) Normal levels of soluble CD25, ferritin, triglycerides, and key HLH-associated cytokines (IFN-γ, IL-10, IL-6).<br>6) Undetectable plasma EBV-DNA.<br>7) For patients with CNS involvement, no evidence of CNS abnormalities.
Partial Response	Meeting all of the following:<br>1) No fever for ≥ 3 days (body temperature < 37.5°C).<br>2) Reduction in splenomegaly on abdominal ultrasound compared with baseline.<br>3) ≥ 50% reduction from baseline in sCD25, ferritin, and key cytokines (IFN- γ, IL- 10, IL- 6).<br>4) ≥ 1-fold increase in absolute neutrophil count (G-CSF support allowed; improvement sustained for ≥ 3 days).<br>5) No progression of other HLH-related symptoms or markers.
No Response	Failure to meet the criteria for partial response.
Relapse	Recurrence of three or more HLH-related symptoms, signs, or laboratory abnormalities after complete remission has been achieved.

HLH, hemophagocytic lymphohistiocytosis; CNS, central nervous system.

Table S3 Proportional hazards assumption check for the Cox regression model.

Variable	chisq	df	*P*
pEBV-DNA.w1	1.780	1	0.182
pEBV-DNA.w2	4.279	1	0.059
ΔFerritin.w1	1.107	1	0.293
ΔFerritin.w2	2.953	1	0.086
ΔIFN-γ.w2	0.685	1	0.408
GLOBAL	8.124	6	0.229

TABLE S4 The variable distribution of the missing data

Variables	Miss.freq	Miss.percentage (%)
Ferritin.d0	0	0
Ferritin.w1	0	0
Ferritin.w2	1	**1.6**
IFN-γ.d0	0	0
IFN-γ.w1	2	3.3
IFN-γ.w2	1	**1.6**
pEBV-DNA.d0	0	0
pEBV-DNA.w1	0	0
pEBV-DNA.w2	0	**0**
Status	0	0
Survival	0	0
Time (month)	0	0

d, day; w, week; freq, frequency.

TABLE S5 Biomarker levels and relative change values (Δ) in patients with favorable and unfavorable outcomes.

**Biomarker**	**Group**	**Baseline (Median, IQR)**		**Week 1 (Median, IQR)**		**Week 2 (Median, IQR)**		**Week 1(Δ values) (Median, IQR)**		**Week 2(Δ values) (Median, IQR)**	
Ferritin	Favorable<br>n = 44	6392.5<br>(1616.5, 11797.2)	*P* = 0.124	680.8<br>(273.8, 2516.2)	*P* = 0.756	188.5<br>(102.8, 596.3)	*P* = **0.020**	−0.8<br>(−0.9, −0.5)	*P* = 0.129	−0.9<br>(−1.0, −0.9)	***P* = 0.004**
	Unfavorable<br>n = 16	2001.0<br>(663.6, 5138.8)		715.6<br>(507.3, 2287.5)		567.9<br>(296.4, 1240.5)		−0.6<br>(−0.9, 0.0)		−0.8<br>(−0.9, 0.3)	
IFN-γ	Favorable<br>n = 44	36.9<br>(3.0, 175.8)	*P* = 0.389	0.9<br>(0.0, 3.1)	***P* = 0.016**	1.0<br>(0.1, 2.6)	*P* = **0.002**	−1.0<br>(−1.0, −1.0)	*P* = 0.009	−1.0<br>(−1.0, −0.7)	***P* = 0.042**
	Unfavorable<br>n = 16	56.0<br>(20.0, 174.7)		5.4<br>(1.5, 31.2)		8.9<br>(1.5, 28.2)		−0.8<br>(−1.0, −0.4)		−0.8<br>(−1.0, 0.2)	

Δ = (level at the end of week 1 or 2−diagnostic level)/diagnostic level

TABLE S6 Univariate Cox regression analysis for predictors of event-free survival in pediatric EBV-HLH patients

**Variable**	**HR (95%CI)**	***P-value***
Age (years)		
< 5	1(ref)	
≥ 5	0.49 (0.14,1.74)	0.271
Gender		
male	1(ref)	
female	0.98 (0.36, 2.70)	0.972
WBC ($\times 10^9$/L)		
< 4	1(ref)	
≥ 4	0.96 (0.36, 2.57)	0.928
Hb (g/L)		
< 90	1(ref)	
≥ 90	0.58 (0.22, 1.55)	0.276
Plt ($\times 10^9$/L)		
< 100	1(ref)	
≥ 100	0.56 (0.16, 1.96)	0.363
TG (mmol/L)		
< 3	1(ref)	
≥ 3	0.82 (0.31, 2.21)	0.698
Fib (g/L)		
< 1.5	1(ref)	
≥ 1.5	2.16 (0.78, 5.95)	0.137
ΔsCD25		
w1	2.3 (0.51, 10.47)	0.281
w2	1.1 (0.67, 1.81)	0.708
w1: low- vs high-decrease	4.11 (0.93, 18.11)	0.062
w2: low- vs high-decrease	4.26 (1.47, 12.35)	0.054
pEBV-DNA		
w1: Positive vs Negative	3.23 (1.17, 8.93)	**0.023**
w2: Positive vs Negative	6.47 (2.31, 18.11)	**<0.001**
ΔFerritin		
w1	2.43 (1.03, 5.74)	**0.047**
w2	2.07 (1.40, 3.06)	**<0.001**
w1: low- vs high-decrease	3.25 (1.18, 8.95)	**0.023**
w2: low- vs high-decrease	7.11 (1.60, 31.57)	**0.010**
ΔIFN-γ		
w1	1.29 (1.08, 1.53)	**0.004**
w2	1.25 (1.09, 1.45)	**0.002**
w1: low- vs high-decrease	4.41 (1.53, 12.7)	**0.006**
w2: low- vs high-decrease	7.03 (2.49, 19.89)	**<0.001**

Δ = (level at the end of week 1 or 2−diagnostic level)/diagnostic level, w1, at the end of week 1,w2, at the end of week 2,

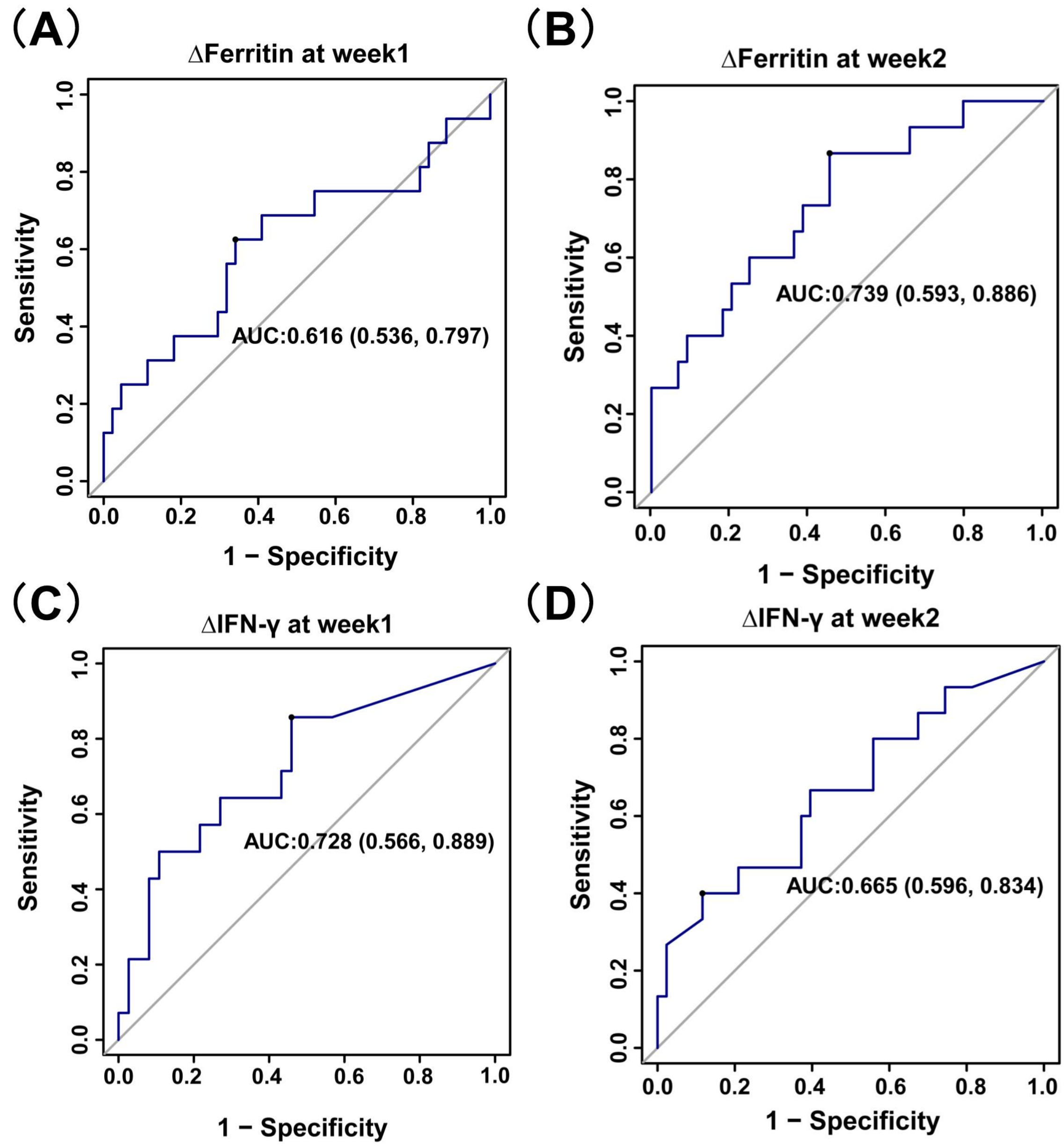

FIGURE S1 Receiver operating characteristic curve analysis of changes in ferritin and IFN-γ levels at the end of the 1st and 2nd weeks of treatment.

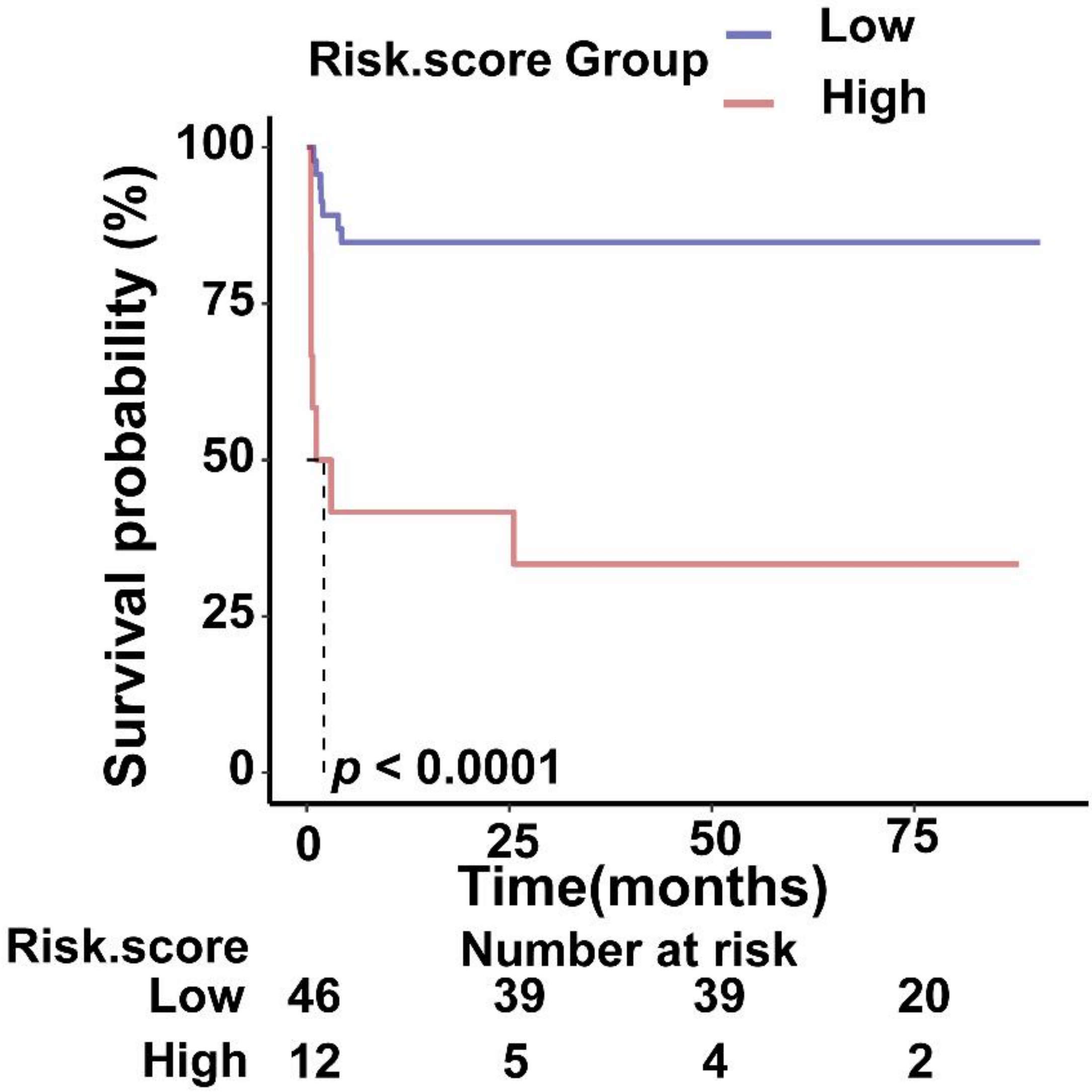

FIGURE S2 Based on the model's risk score, EBV-HLH patients were stratified into high-risk and low-risk groups, demonstrating a statistically significant difference between the groups ($P < 0.0001$).
